# Evidence of at Least Two Introductions of HIV-1 in the Amerindian Warao Population from Venezuela

**DOI:** 10.1371/journal.pone.0040626

**Published:** 2012-07-12

**Authors:** Héctor R. Rangel, Mailis Maes, Julian Villalba, Yoneira Sulbarán, Jacobus H. de Waard, Gonzalo Bello, Flor H. Pujol

**Affiliations:** 1 Laboratorio de Virología Molecular, Centro de Microbiología y Biología Celular, Instituto Venezolano de Investigaciones Científicas, Caracas, Venezuela; 2 Laboratorio de Tuberculosis, Instituto de Biomedicina, Universidad Central de Venezuela, Caracas, Venezuela; 3 Laboratorio de AIDS & Imunologia Molecular, Instituto Oswaldo Cruz – FIOCRUZ, Rio de Janeiro, Brazil; University of Florida, United States of America

## Abstract

**Background:**

The Venezuelan Amerindians were, until recently, free of human immunodeficiency virus (HIV) infection. However, in 2007, HIV-1 infection was detected for the first time in the Warao Amerindian population living in the Eastern part of Venezuela, in the delta of the Orinoco river. The aim of this study was to analyze the genetic diversity of the HIV-1 circulating in this population.

**Methodology/Principal Findings:**

The *pol* genomic region was sequenced for 16 HIV-1 isolates and for some of them, sequences from *env*, *vif* and *nef* genomic regions were obtained. All HIV-1 isolates were classified as subtype B, with exception of one that was classified as subtype C. The 15 subtype B isolates exhibited a high degree of genetic similarity and formed a highly supported monophyletic cluster in each genomic region analyzed. Evolutionary analyses of the *pol* genomic region indicated that the date of the most recent common ancestor of the Waraos subtype B clade dates back to the late 1990s.

**Conclusions/Significance:**

At least two independent introductions of HIV-1 have occurred in the Warao Amerindians from Venezuela. The HIV-1 subtype B was successfully established and got disseminated in the community, while no evidence of local dissemination of the HIV-1 subtype C was detected in this study. These results warrant further surveys to evaluate the burden of this disease, which can be particularly devastating in this Amerindian population, with a high prevalence of tuberculosis, hepatitis B, among other infectious diseases, and with limited access to primary health care.

## Introduction

According to the UNAIDS Global 2010 report, Human immunodeficiency virus type 1 (HIV-1) infects around 33 million people worldwide [1]. Estimates predict that around 1.7 million are infected in Latin America [2]. In Venezuela, HIV-1 prevalence is estimated at approximately 0.7% [3]. HIV-1 subtype B is the most prevalent subtype in the Americas and particularly in Venezuela [2], [4].

Amerindians, especially those living in remote communities in difficult access areas, are generally not infected with HIV. However, recent reports have documented HIV infection in some Amerindian communities in the Amazon basin [5]–[8]. The Warao Amerindians live in the Eastern part of Venezuela, were the Orinoco river forms a wide delta that branches off into hundreds of rivers and waterways that flow through 41.000 Km^2^ of swampy forests [9]. High prevalence of hepatitis B virus (HBV) infection and tuberculosis is found among the Waraos [10], [11]. Tuberculin PPD surveys have shown that approximately 60% of the population has been infected with tuberculosis (de Waard, JH, personal communication). In 2007, the Venezuelan Red Cross diagnosed for the first time HIV-1 infection in this population, reporting 5 cases. In this study we report the molecular characterization of the HIV-1 strains circulating within the Warao Amerindians two years after the first cases were detected.

## Results

**Figure 1:**
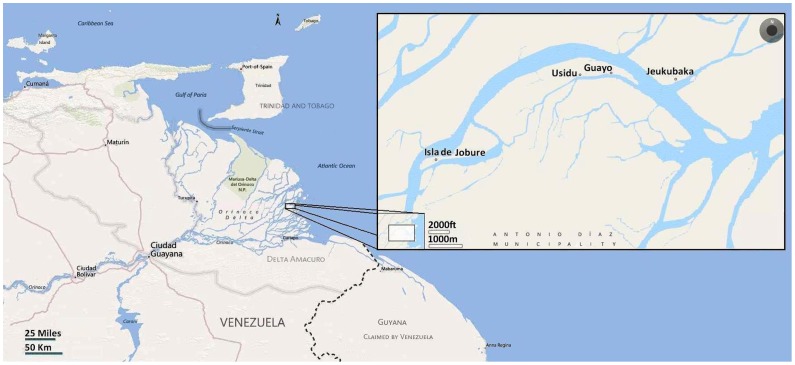
Map of the Orinoco Delta showing the location of the Warao communities studied.

**Figure 2:**
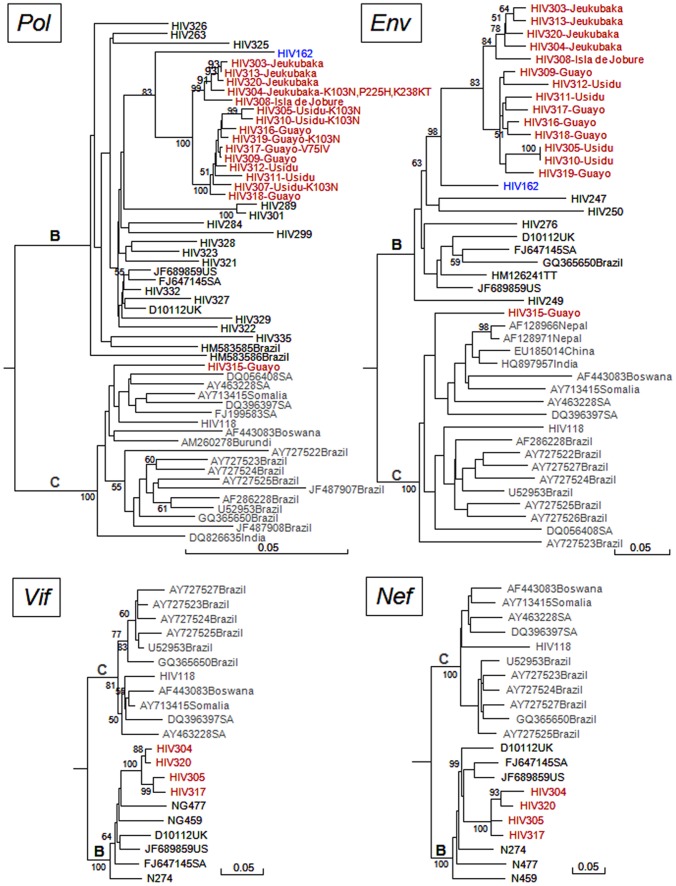
Phylogenetic analysis of HIV isolates infecting Waraos (Neighbor Joining method). Pol region (approximately 1400 nt). **Env region** (approximately 280 nt). **Vif region** (approximately 540 nt). **Nef region** (approximately 780 nt). Isolates are designated by their GenBank accession number and the name of the country of origin, except for Venezuelan isolates, which are shown by their name and in red for the ones infecting Waraos. The locality of origin of each Warao individual. RT mutations are shown in the name when present in the sequence. HIV162 sequence, which appears to share a common ancestor with the Amerindian subtype B clade, is shown in blue. Subtype C sequences are shown in grey. Phylogenetic analysis was performed by Neighbor Joining and distance calculated with Kimura 2 parameters. Bootstrap values over 50% are shown in the trees. Letters in bold in the tree indicate subtype.

By 2009, 32 identified patients infected with HIV-1 were living in Antonio Diaz Municipality in the Orinoco Delta [Maes, M, personal communication]. Plasma of 18 patients, living in 4 communities (Figure 1), was available for molecular studies and in 16/18 the sequence of *pol* genomic region could be analyzed. Phylogenetic analysis of *pol* region showed that 15 out of 16 HIV-1 strains belonged to subtype B and the remaining strain to subtype C, a subtype rarely found in Venezuela [4] (Figure 2). All the subtype B strains were found infecting men, while the subtype C strains was found in one woman.

The subtype B sequences from Waraos formed a highly supported monophyletic clade in each genomic region analyzed (Figure 2). At the *pol* genomic region, the subtype B strains infecting Waraos displayed 97–99% identity between them and only 94–96% identity with the closely related sequence available at GenBank (accession number D10112). A sequence from a Venezuelan patient from Maracay, a city distant from the Orinoco Delta (HIV162, accession number FJ65968) was found to share an ancestor with Waraós subtype B clade, exhibiting 94–95% identity with this clade at the *pol* genomic region. Phylogenetic analyses of the *pol*, *env* and *vif* genomic regions indicated that the Waraos clade was subdivided in two well supported subclades. Strains form individuals living in a same community were grouped in the same subclade (Figure 2). The only HIV-1 subtype C isolate found among Waraos was more closely related to the African and Asian strains (closely related sequences available at GenBank), than to the Brazilian subtype C clade. However, the grouping with these strains was not supported by a high bootstrap value (Figure 2).

**Figure 3:**
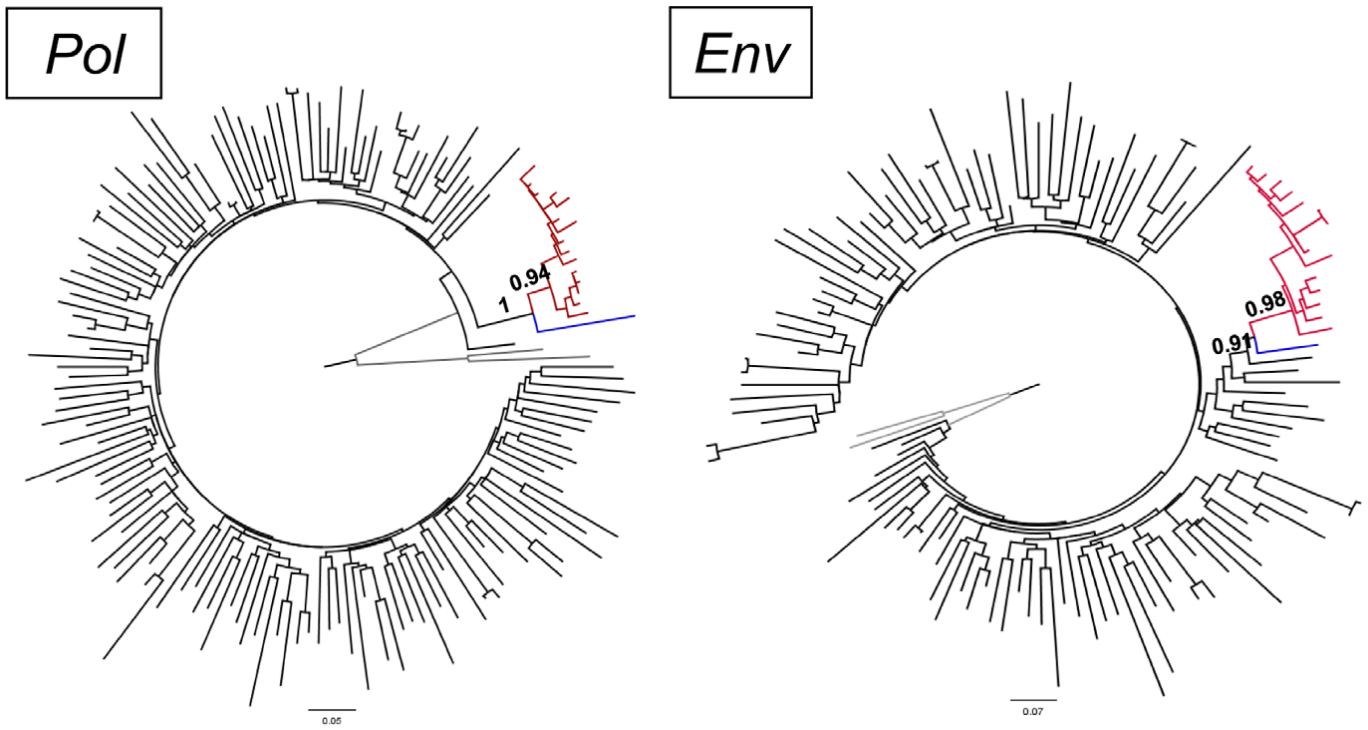
Maximum likelihood tree of the *pol* (approximately 1400 nt) and *env* (approximately 280 nt) genes of HIV-1 subtype B strains circulating in Venezuela. HIV-1 subtype B sequences infecting Waraos are shown in red while sequence from patient 162 is shown in blue. The aLRT support values are indicated only at key nodes. The tree was rooted using subtype C reference strains (grey branches) as outgroups. Horizontal branch lengths are drawn to scale with the bar at the bottom indicating nucleotide substitutions per site.

In order to confirm the hypothesis of a single introduction of the HIV-1 subtype B in the Warao Amerindians, Maximum Likelihood (ML) phylogenetic analysis was performed. HIV-1 sequences from Waraos were analyzed with a set of 141 *pol* and 112 *env* subtype B “background” sequences isolated from the general population in Venezuela. The ML tree confirmed the monophyletic clustering of the subtype B sequences from Waraos and also supported the relative close phylogenetic relationship between the Warao clade and the Venezuelan sequence HIV162, both when analyzing the *pol* and the *env* regions (Figure 3).

**Figure 4:**
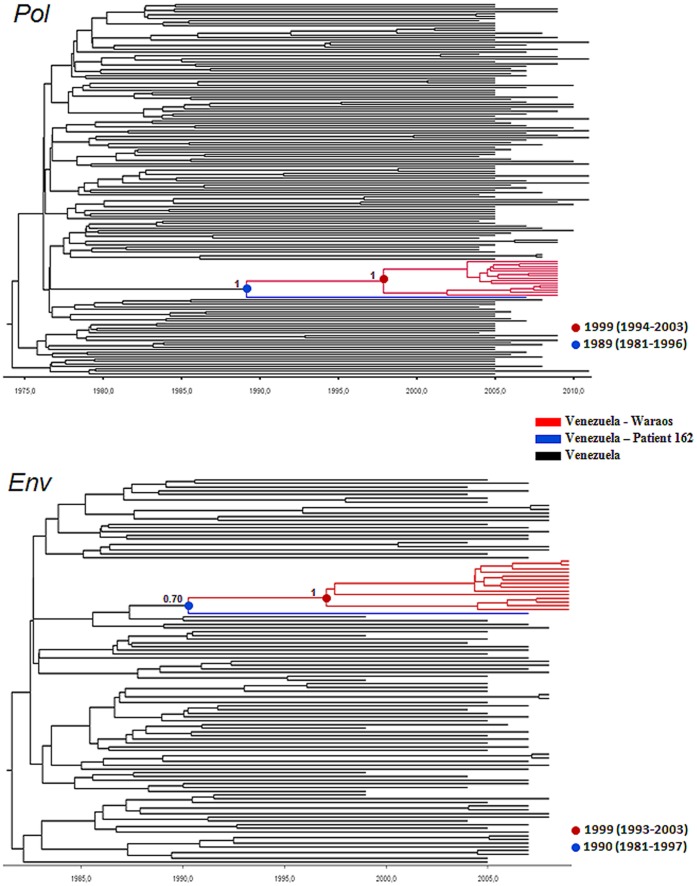
Time-scaled Bayesian Maximum Clade Credibility tree of the *pol* (approximately 1400 nt) and *env* (approximately 280 nt) genes of HIV-1 subtype B strains circulating in Venezuela. HIV-1 subtype B sequences infecting Waraos are shown in red while sequence from patient 162 is shown in blue The *PP* support and the age (with 95% High Posterior Density interval in parentheses) of key nodes corresponding to the most recent common ancestors of Waraos (red node) and Waraos/Venezuelan patient 162 (blue node) strains are shown. Branch lengths of the trees correspond to length of time (see the time scale bar). The tree is automatically rooted under the assumption of a relaxed molecular clock.

Estimates of the time of the most recent common ancestor (*T*mrca, years) of subtype B Warao clade were obtained using a Bayesian MCMC approach. A total of 156 *pol* sequences (collected between 2004 and 2011) and 104 *env* sequences from Venezuela (isolates collected between 1999 and 2009) were used for this analysis. The 95% HPD intervals of the estimated rate of evolution of both *pol* (1.6–2.5×10^−3^ subst./site/year) and *env* (4.1–7.8×10^−3^ subst./site/year) datasets almost coincide with the informative prior interval used in our analyses, indicating that not much temporal information was added by the data. Meanwhile, the coefficients of rate variation of both *pol* (0.27; 95% HPD: 0.22–0.33) and *env* (0.30; 95% HPD: 0.22–0.40) genomic regions were higher than zero, thus demonstrating a significant variation of substitution rate among branches and supporting the use of a relaxed molecular clock model. The *T*mrca of the Warao subtype B clade was estimated at 1999 (95% HPD: 1993–2003) when analyzing both *pol* and *env* genomic regions; while the *T*mrca of the Waraos and Venezuelan patient HIV162 was 10 years older (Figure 4). The *T*mrca of all subtype B sequences from the general population in Venezuela was estimated at 1977 (95% HPD: 1968–1984) and 1979 (95% HPD: 1968–1986), according to the analysis of the *pol* and *env* regions, respectively.

Six out of 15 subtype B strains from the Waraós clade exhibited drug resistance mutations to NRTIs or NNRTIs. The NRTI resistance mutation V75IV and the NNRTI resistance mutations P225H and K238KT were found in one strain each, while the NNRTI resistance mutation K103N was detected in five strains, and found as a single mutation in the Guayo-Usidu subclade (Figure 2). Not all the individuals infected with drug-resistant viruses received treatment before this study. The subtype C strain did not harbor any resistance mutation.

## Discussion

HIV-1 infection in Waraos was first reported by the Venezuelan Red Cross and local health authorities, who diagnosed the first cases in 2007 in symptomatic patients. Our study two years later indicates that at least two independent introductions of HIV-1 occurred in the Venezuelan Warao population: a single introduction of a subtype B strain and a HIV-1 subtype C introduction, documented in an infected woman. The Warao subtype B clade split into two subclusters indicating two independent networks of viral dissemination within the communities. One of the subclades consisted of patients from the communities of San Francisco de Guayo and Usidu, and the other subclade included patients of a community called Jeukubaka and one isolated patient from a community called Isla de Jobure. All the subtype B cases were found among men who refered sex with men. Sex practices between men have been documented among Warao Amerindians in Venezuela [12], [13].

HIV-1 subtype B is highly predominant in Venezuela [4]. Indeed, the sequence from another Venezuelan patient, HIV162, was found to share a common ancestor with the Waraós clade. This man, with heterosexual habits, who lives far away from the Orinoco Delta, and denied having visited this region, might have been in contact with the person or partners of the person that might have transmitted HIV-1 subtype B to the first Warao(s) infected with this subtype. Bayesian coalescence analyses suggested that subtype B clade probably started to disseminate among Waraos at around 1999, a date compatible with the appearance of immunodeficiency symptoms in some Warao individuals in 2007. The Venezuelan patient HIV162 share a relative distant common ancestor with the Waraos subtype B clade estimated at around 1989–1990. Thus, despite patient HIV162 and Waraos could be part of the same network of HIV transmission in Venezuela, a direct epidemiological link between that Venezuelan patient and the Warao community was discarded.

The Bayesian Coalescence analyses estimated the *T*mrca of subtype B strains circulating in Venezuela between the mid and the late 1970s, a date compatible with the identification of the first AIDS case in the country in 1985. However, whether this estimated *T*mrca represents a good approximation to the onset date of the subtype B epidemic in Venezuela is unclear because the monophyletic or polyphyletic origin of Venezuelan subtype B epidemic has not been formally tested until now.

In contrast to subtype B, HIV-1 subtype C is still very rare in Venezuela [8]. The woman infected with HIV-1 subtype C denied any sexual contact apart from her husband, who died one year ago with cachexia and chronic diarrhea, both symptoms characteristic of the CDC staging system for AIDS [14]. The serum sample of her husband was not available for HIV molecular testing, but was found seropositive before this study. The presence of HIV-1 subtype C infection in a Warao might be due to a contact with a person not living in Venezuela. The subtype C isolate infecting Waraos did not group with Brazilian clade but rather with Nepalese and Chinese subtype C isolates. Foreign strains of *Mycobacterium tuberculosis*, circulating in India and Philippines, have been detected earlier in the Warao population [15], but have not been found in other localities in Venezuela [16], suggesting a contact with this part of the world. We speculate that the presence of this viral subtype in this geographical area might be related to a trade route of Asian ships that travel through the Orinoco Delta to transport metals from upstream mining industries. A Guyanese origin (whose border is adjacent to Orinoco Delta) cannot be ruled out either. The few HIV-1 Guyanese isolates that have been analyzed belong to subtype B [17]. The Guyanese subtype B isolates were not closely related to the Warao subtype B clade (data not shown). Guyana has the highest prevalence of HIV in South America (1.55%) [1], [18], which suggests that a more diverse subtype distribution in this country might be probable.

Many of the HIV-1 subtype B isolates from this study exhibited RT drug resistance mutations, and some of them without history of receiving HAART. It would then be advisable to suppose that these resistance mutations might be present in the virus infecting other individuals. An effective treatment program for this particular community should be considered. In addition, there is a serious concern on the difficulty of Amerindians to comply to HAART treatment, in order to achieve an adequate adherence. As reported for other Amerindians, there is a poor knowledge about HIV infection and low acceptance of prevention and treatment [5], [8]. Emerging prevention strategies developed in the US for Native Americans [19] may be evaluated and adapted to this particular ethnic group.

In conclusion, at least two independent introductions of HIV-1 have occurred in Warao Amerindians from Venezuela. The subtype B introduction seems to have disseminated within the community, perhaps through homosexual relations. More studies are needed to estimate the burden of this devastating infection in such a vulnerable community, with limited access to primary health care.

## Materials and Methods

### Ethic Statements

Plasma samples from Warao individuals who previously signed a written informed consent were collected. Illiterate individuals who wanted to participate in the study put their fingerprint instead the signature. This study was approved by the ethical commission of Instituto de Biomedicina in Caracas, Venezuela. The Venezuelan Ministry of Health and the National and Regional HIV/AIDS programmes received reports of this study previous to the publication.

### Study Population

Blood (3–4 ml) from 18 HIV-1 infected Warao Amerindians, previously diagnosed as anti-HIV-1 positive in population surveys conducted on symptomatic patients, was collected and plasma separated in the field. The individuals belonged to four communities, San Francisco de Guayo, Usidu, Jeukubaka and Isla de Jobure (Figure 1). The sample group consisted of 17 men and one woman, 8 of them with previously antiretroviral drug treatment. *Pol* partial genomic sequence was available for 16 and *env*, *vif* or *nef* sequences for some isolates, according to previous reported procedures [4]. Nucleotide sequence data have been deposited into the GenBank database under the accession numbers JQ689592–JQ689659.

### Subtyping

Subtype and genotypic resistance evaluation were performed by submitting sequences to the Stanford University HIV Drug Resistance Database (http://hivdb.stanford.edu/index.html) and by phylogenetic inference. BLAST analysis was also carried out to identify the most closely related sequences in the GenBank.

### Sequence Alignments

The HIV-1 *pol* (*n* = 16), *env* (*n* = 15), *nef* and *vif* (n = 4) sequences obtained from the Waraos were aligned against a set of Venezuelan sequences and related sequences obtained from BLAST analysis, for phylogenetic analysis by Neighbor Joining Method, and against 141 *pol* and 112 *env* subtype B “background” sequences isolated from the general population in Venezuela for maximum likelihood (ML) and Bayesian analysis. Subtype B “background” Venezuelan sequences were gathered from the Los Alamos HIV Database (http://hiv-web.lanl.gov/). Sequences were excluded if they originated from the same patient or displayed a multidrug resistance profile. In order to avoid any bias on the phylogenetic analyses, a few sites with major antiretroviral drug resistance mutations and other substitutions associated to drug resistance in PR (46) and RT (70, 90, 103, 106, 138, 179, 184), in at least two sequences, were also excluded. Nucleotide sequences were aligned using CLUSTAL X program [20]. The first alignment of approximately 1000-nt covered the entire protease and part of the reverse transcriptase (PR/RT) region of *pol* gene (positions 2253–3520 relative to HXB2). The second alignment of approximately 310-nt spanned the V3 region of the E*nv*-gp120 gene (positions 7023 to 7330 relative to HXB2). For *nef* and *vif*, alignements spanned almost complete genes (positions 4980 to 5520 and 8760 to 9450, respectively). Alignments are available from the authors upon request.

### Phylogenetic Analysis

Phylogenetic tree was inferred by Neighbor Joining method using a computerized program DNAman 5.2.2. (Lynnon Bio Soft, Canadá), and the ML method under the GTR+I+Γ_4_ nucleotide substitution model, selected using the jModeltest program [21]. ML tree was reconstructed with program PhyML [22] using an online web server [23]. Heuristic tree search was performed using the SPR branch-swapping algorithm and the reliability of the obtained topology was estimated with the approximate likelihood-ratio test (*aLRT*) [24] based on a Shimodaira-Hasegawa-like procedure. Trees were visualized using the FigTree v1.3.1 program [25].

### Estimation of Evolutionary Rates and Dates

Estimates of the evolutionary rate (μ, units are nucleotide substitutions per site per year, subst./site/year) and the time of the most recent common ancestor (Tmrca, years) were obtained using a Bayesian Markov Chain Monte Carlo (MCMC) approach as implemented in BEAST v1.6.2 [26], [27]. The temporal structure of the *pol* and *env* datasets was not sufficient to reliably estimate the evolutionary rate under a chronological time-scale employing the dates of the sequences. Therefore, the 95% Highest Posterior Density (HPD) intervals of rates of evolution previously estimated for subtype B at PR/RT (1.5×10^−3^–2.5×10^−3^ subst./site/year) [28] and gp120-V3 (4×10^−3^–8×10^−3^ subst./site/year) [29] genomic regions were incorporated as a prior probability distribution in our analyses. Analyses were performed with a Bayesian Skyline coalescent tree prior [30], under the GTR+I+Γ nucleotide substitution model and using a relaxed (uncorrelated Lognormal model) molecular clock [31]. Three separate MCMC chains were run for 20×10^6^ generations with a burn-in of 2×10^6^. BEAST output was analyzed using TRACER v1.4 [32], with uncertainty in parameter estimates reflected in the 95% HPD intervals. All Bayesian MCMC independent runs converged to almost identical values for all parameters, and the ESS values for parameter estimates were >100. The results reported are the combined estimates of the three independent runs. The programs TreeAnnotator v1.6.2 (available from http://beast.bio.ed.ac.uk) and FigTree v1.3.1 were used to summarize the posterior trees samples from BEAST runs and to visualize the annotated time-scaled maximum clade credibility (MCC) trees.
